# Metabotropic Glutamate Receptor 1 Expression and Its Polymorphic Variants Associate with Breast Cancer Phenotypes

**DOI:** 10.1371/journal.pone.0069851

**Published:** 2013-07-26

**Authors:** Madhura S. Mehta, Sonia C. Dolfi, Roman Bronfenbrener, Erhan Bilal, Chunxia Chen, Dirk Moore, Yong Lin, Hussein Rahim, Seena Aisner, Romona D. Kersellius, Jessica Teh, Suzie Chen, Deborah L. Toppmeyer, Dan J. Medina, Shridar Ganesan, Alexei Vazquez, Kim M. Hirshfield

**Affiliations:** 1 Division of Medical Oncology, Department of Medicine, The Cancer Institute of New Jersey/University of Medicine and Dentistry of New Jersey - Robert Wood Johnson Medical School, Piscataway, New Jersey, United States of America; 2 Department of Biometrics, The Cancer Institute of New Jersey/University of Medicine and Dentistry of New Jersey - Robert Wood Johnson Medical School, Piscataway, New Jersey, United States of America; 3 Department of Pathology and Laboratory Medicine, University of Medicine and Dentistry of New Jersey - New Jersey Medical School, Newark, New Jersey, United States of America; 4 Department of Chemical Biology, Rutgers, The State University of New Jersey, Piscataway, New Jersey, United States of America; 5 Department of Radiation Oncology, The Cancer Institute of New Jersey/University of Medicine and Dentistry of New Jersey- Robert Wood Johnson Medical School, Piscataway, New Jersey, United States of America; University of Manitoba, Canada

## Abstract

Several epidemiological studies have suggested a link between melanoma and breast cancer. Metabotropic glutamate receptor 1 (GRM1), which is involved in many cellular processes including proliferation and differentiation, has been implicated in melanomagenesis, with ectopic expression of GRM1 causing malignant transformation of melanocytes. This study was undertaken to evaluate GRM1 expression and polymorphic variants in GRM1 for associations with breast cancer phenotypes. Three single nucleotide polymorphisms (SNPs) in GRM1 were evaluated for associations with breast cancer clinicopathologic variables. GRM1 expression was evaluated in human normal and cancerous breast tissue and for *in vitro* response to hormonal manipulation. Genotyping was performed on genomic DNA from over 1,000 breast cancer patients. Rs6923492 and rs362962 genotypes associated with age at diagnosis that was highly dependent upon the breast cancer molecular phenotype. The rs362962 TT genotype also associated with risk of estrogen receptor or progesterone receptor positive breast cancer. *In vitro* analysis showed increased GRM1 expression in breast cancer cells treated with estrogen or the combination of estrogen and progesterone, but reduced GRM1 expression with tamoxifen treatment. Evaluation of GRM1 expression in human breast tumor specimens demonstrated significant correlations between GRM1 staining with tissue type and molecular features. Furthermore, analysis of gene expression data from primary breast tumors showed that high GRM1 expression correlated with a shorter distant metastasis-free survival as compared to low GRM1 expression in tamoxifen-treated patients. Additionally, induced knockdown of GRM1 in an estrogen receptor positive breast cancer cell line correlated with reduced cell proliferation. Taken together, these findings suggest a functional role for GRM1 in breast cancer.

## Introduction

Over 230,000 new breast cancer cases are estimated to be diagnosed in 2013, making breast cancer one of the most frequently diagnosed cancers in women [1]. Many factors including gender, age, family history, and gynecologic history are associated with breast cancer risk. However, in high-risk families, these breast cancer risk factors may be secondary to mutations in the breast cancer associated (BRCA) genes, *i.e.* BRCA1 and BRCA2. Despite characterization of these genetic susceptibility genes, fewer than 10% of all breast cancers are attributable to mutations in BRCA1/BRCA2 [2]. More likely the majority of breast cancer is polygenic and caused by low penetrance, high frequency polymorphisms, such as single nucleotide polymorphisms (SNPs) [3]. Many studies have implicated SNPs in risk and age at diagnosis of breast cancer. SNPs, particularly those in genes involved in pathways integral in tumorigenesis, such as cell growth, DNA repair, cell death and cell proliferation, and estrogen metabolism are likely involved in risk for the majority of breast cancers [3]-[8]. Though epidemiologic studies have linked the risk of developing melanoma and breast cancer, it is not related to known genetic susceptibility mutations for either disease [9]. A single case-control study evaluated three SNPs in metabotropic glutamate receptor 1 (GRM1) for their association with risk of developing melanoma [10]. This study found that carriers of the C allele of GRM1 rs362962 had a higher risk of developing melanoma and that the difference became greater in a subgroup of patients with a low level of sun exposure and with tumors located on the trunk and extremities. No studies have evaluated SNPs in GRM1 and risk of breast cancer.

GRM1, a member of the G-protein coupled receptor (GPCR) family, is most notably known for its role in nervous system development, function, and pathology [11]-[15]. However, its significance in other organ systems has been subsequently demonstrated, particularly in regard to tumorigenesis [16]-[20]. Insertional mutagenesis resulting in disruption in intron 3 of GRM1 induced its expression and unexpectedly produced melanomas with 100% penetrance in a mouse model [16]. The role of GRM1 in melanomagenesis has been subsequently elucidated, while melanoma growth suppression occurs with its targeted inhibition [21]-[24]. GRM1 is expressed in many cancer cell types as compared to normal counterparts underscoring its potential role in tumor behavior [15], [19], [25], [26]. Cancer cell lines have also been observed to secrete its ligand glutamate extracellularly where it may act in an autocrine or paracrine manner to activate GRM1. The secreted glutamate has been hypothesized to promote cancer progression and to modulate tumor microenvironment [25].

Characterization of the intracellular signals evoked as a consequence of GRM1 activation provides the basis for its molecular role in oncogenesis and tumor progression [27], [28]. Glutamate-induced activation of GRM1 leads to interactions with G proteins and initiation of a cascade of intracellular signaling that results in pro-proliferation, pro-survival, and anti-apoptotic signals. Intracellular signaling occurs through phosphorylation and activation of the MAPK and AKT pathways and release of intracellular calcium [14], [23], [27]-[29]. Likewise, modulation of GRM1 activity by GRM1 modulators riluzole and BAY36-7620 or silencing RNA inhibits anchorage independent growth, migration, invasion, proliferation, and downstream phosphorylation/activation of AKT and ERK *in vitro* [19], [21], [23], [24]. GRM1 modulation also affects *in vivo* tumorigenesis of melanoma, prostate and renal cancer cells [19], [24], [26]. Aberrations in the MAPK pathway have been reported in breast cancer though the mechanism is not well understood. However, it is known that increased MAPK activity is correlated with reduced disease free survival in patients receiving tamoxifen [30].

As the role of GRM1 continues to be elucidated in melanoma, less is known about its role in breast cancer. Recently, Speyer *et al.* [31] have demonstrated that activation of GRM1 with L-quisqualate results in phosphorylation of AKT in estrogen receptor negative (ER-) breast cancer cells, that this phosphorylation can be abrogated through pre-treatment with a GRM1 inhibitor, and that GRM1 inhibition results in reduced cell growth *in vitro* and *in vivo*. In this only study describing GRM1 in breast cancer, ER+ breast cancer cells were not evaluated [31]. Therefore, we undertook a study to evaluate SNPs in GRM1 for correlation with development of specific breast cancer molecular subtypes and age at diagnosis. We also evaluated GRM1 expression in human breast cancer, as a function of hormonal modulation, and for its association with risk of breast cancer recurrence.

## Materials and Methods

### Study Subjects

Patients were invited to participate in a protocol that supports gene association studies from 2004-2009 through the Stacy Goldstein Breast Cancer Center at The Cancer Institute of New Jersey (CINJ). Greater than 95% of eligible individuals gave consent for participation. Eligibility included a history of biopsy-proven breast cancer verified by pathology records and confirmed on review by the CINJ institutional breast pathologist. In fewer than 5% of cases, slides were not available for review and pathological features were based on available pathology reports from other institutions. Venipuncture was performed to obtain 5mL blood. Medical records were abstracted for clinical information. Negative estrogen receptor (ER) and progesterone receptor (PR) staining were defined by immunohistochemical (IHC) staining of <10%. BRCA1/BRCA2 testing was performed where clinically indicated and patients with known BRCA1/BRCA2 mutations were then excluded from age at diagnosis analysis due to potential confounding bias. Lobular carcinoma *in situ* (LCIS) was excluded for all analyses. Investigations were performed with prior approval by the University of Medicine and Dentistry of New Jersey Institutional Review Board (IRB). Written consent was obtained using the IRB-approved consent form.

### Candidate GRM1 SNPs

GRM1 SNPs were selected to study genotype-specific associations with breast cancer phenotypes: rs6923492 (non-synonymous, Ex10+341C>T), rs362962 (IVS4, c.1186+7836T>C), and rs1125462 (IVS2, c.701-62652A>G). SNPs were selected based on the following selection criteria: location of the SNP, previous positive association in a melanoma case-control study of GRM1 SNPs [10], predicted functional role based on amino acid change, and genotype frequency distributions. GRM1 rs854145, which was investigated by Ortiz *et al.* [10], was excluded because it did not prove to be significant for any associations with melanoma and its suboptimal allelic and genotypic frequency distributions leading to low power for detecting associations. Instead, GRM1 rs1125462 was analyzed in this study. The Genome Variation Server (sponsored by the Seattle SNP group, http://gvs.gs.washington.edu/GVS/), identified that rs1125462 represented a SNP from a linkage disequilibrium (LD) block distinct from the other two SNPs, had a high minor allele frequency (47% in the HapMap CEPH Caucasian population) and was in a different region of the gene from the other two SNPs. As no associations were observed for rs1125462, no data is shown for this locus.

### Genotyping

Using 1 mL of peripheral blood obtained through venipuncture, genomic DNA was extracted using a spin column-based method according to manufacturer protocol (QIAGEN, Valencia, CA). Genotyping was performed using Taqman assays on the ABI 7900HT Fast Real-Time PCR System (Applied Biosystems, Foster City, CA). Briefly, reactions were performed using 5-10 ng genomic DNA in 10 µL volume with these PCR cycling conditions: 50°C for 2 minutes, 95°C for 10 minutes, followed by 40 cycles at 95°C for 15 seconds and 60°C for 1 minute. Fewer than 1% of samples failed genotyping. Duplicative genotyping was performed in 10% of samples and a subset underwent direct sequencing.

### Hormone and Drug Manipulation of Breast Cancer Cell Lines

MCF7 (ER+/PR+) and MDA-MB-231 (ER−/PR-) breast cancer cells were seeded in 6 well plates in DMEM containing 10% FBS. Where indicated, media was changed at 24 h to phenol free DMEM containing 10% charcoal-stripped FBS (PF-DMEM). After 72 h incubation in PF-DMEM, cells were treated with 50 nM 17β-estradiol (E2), 100 nM progesterone (P), 100 nM 4-hydroxytamoxifen (T), or a combination of hormonal agents. Cells were retreated at 24 h with exchange of media and drug. After 48 h, cells were washed with 1X PBS, removed from the plate, and spun down at 1500 g for 5 min. Cell lysates were prepared in RIPA buffer with 1% protease inhibitor cocktail. Protein concentration was determined by Bradford assay (Bio-Rad, Hercules, CA). Lysates (20 ug of protein) were resolved on a 4-15% SDS-PAGE gradient gel followed by transfer to PVDF membrane. Membranes were incubated in blocking buffer consisting of 5% powdered milk in PBS+0.1% Tween 20 at room temperature for 1 h. Blocked membranes were immunoblotted with GRM1 primary antibody (Novus Biologicals, Littleton, CO) at 1∶2000 dilution in blocking buffer overnight at 4°C and β actin primary antibody (Sigma Aldrich, St. Louis, MO) at 1∶10,000 dilution in blocking buffer for 40 min at room temperature. Detection by enzyme-linked chemiluminescence was performed according to manufacturer protocol (ECL; Pierce Biotechnology Inc., Rockford, IL). Western blots from three independent experiments were quantitated using the ImageJ program, and relative GRM1 protein levels were calculated after normalization to β-actin.

### GRM1 Inducible Knockdown and Cell Proliferation Assays

To generate MCF7 cells stably expressing a doxycycline-inducible siGRM1 expression vector, TetR plasmid (neomycin-resistant) was co-transfected with Zeocin plasmid and TetR clones were selected with Zeocin (Life Technologies, Grand Island, NY) at a concentration of 300 µg/ml. siGrm1 sequence was cloned into the inducible siRNA expression vector pRNATin-H1.1/Hygro (GenScript, Piscataway, NJ, USA). Stable siRNA/TetR-transfected MCF7-siGRM1 clones were selected in neomycin 300 µg/ml and Hygromycin B 50 µg/ml. Two independent MCF7 siGRM1 clones (8-1 and 8-3) were cultured in RPMI growth medium containing 10% FBS, 50 µg/ml Hygromycin B, and 300 µg/ml geneticin. To confirm inducible knockdown of GRM1, cells were grown in complete growth medium containing 4 µg/ml doxycycline (+Dox) for a total of 10 days with fresh medium added to the cells every three days. Control cells were grown in complete growth medium (-Dox). Cell lysates were prepared and western blot was performed and quantitated. GRM1 protein levels were normalized to β-actin.

To determine the effect of GRM1 knockdown on cell number, cells were seeded in 12 well plates and treated the next day with doxycycline-containing medium or complete growth medium for 10 days. Cells were then trypsinized and counted on the Vi-CELL Cell Viability Analyzer (Beckman Coulter, Indianapolis, IN). As an independent method to determine relative cell number between control and GRM1 knockdown, the CellTiter 96® AQueous Non-Radioactive Cell Proliferation Assay was performed per manufacturer instructions (Promega, Madison, WI). Briefly, cells were seeded in 96 well plates in complete growth medium and changed to doxycycline-containing medium or complete growth medium the next day. After a 10-day incubation, MTS/PMS solution was added and absorbance was measured at 490 nm.

### Tissue Microarray Analysis

GRM1 expression was evaluated in a set of four tissue microarrays (TMAs) with no overlapping cases: BR1503, BRC961, BR963, and BR1003 (US BioMax Inc., Rockville, MD). These TMAs had normal, high-risk breast abnormalities, intraductal and invasive breast cancer samples with variable histopathologic annotation, e.g. ER, PR, and Her-2 status, American Joint Committee on Cancer TNM classification for cancer staging, grade. Histology was reviewed on hematoxylin and eosin stain. Tissue IHC was performed for GRM1 using the Ventana Medical Systems *Discovery XT* automated immunostainer. Anti-GRM1 (Abcam, Cat# Ab27192, rabbit polyclonal) was optimized on human control tissues including melanoma. Slides were cut at 4 um, deparaffinized and antigen retrieval was performed using CC1 (Cell Conditioning 1, Ventana Medical Systems, Cat #950-124). Pre-dilute anti-GRM1 antibody was applied at a dilution of 1∶2 and incubated at 37°C for 1 hour. Donkey anti-rabbit secondary antibody (Jackson Immunolab, Cat# 711-065-152) was applied at 1∶500 and incubated at 37°C for 1 hour, followed by chromogenic detection kit DABMap (Ventana Medical Systems, Cat #760-124). Slides were counterstained with hematoxylin and dehydrated and cleared before cover-slipping from xylene.

TMAs were stained, read, scanned, and digital images made for review by the study pathologist. Fibroadenomas were excluded from analyses since they represent neither normal tissue nor a breast cancer. Similarly, atypical ductal hyperplasia, atypical lobular hyperplasia and lobular carcinoma in situ samples were excluded from analyses as they represent high-risk breast abnormalities. While stratification by high-risk breast abnormalities was attempted, there were too few samples for association analyses. For IHC analysis, samples represented as “cancer” included intraductal carcinoma, invasive ductal and invasive lobular carcinoma only. Samples represented as “normal” only included benign, normal tissue, and typical hyperplasia. IHC analysis of the TMA for membrane staining of GRM1 was considered “negative” for samples that were scored as 0, and “positive” for samples with a score of 1+ to 3+.

### Recurrence of Tamoxifen-treated Breast Cancer as a Function of GRM1 Expression

The public breast cancer gene expression dataset from Loi *et al.* [32] was obtained from GEO (http://www.ncbi.nlm.nih.gov/geo/query/acc.cgi?acc=GSE6532) and reanalyzed. Briefly, gene expression from 268 primary ER+ breast tumors where patients received adjuvant tamoxifen monotherapy were re-normalized as follows: Raw Affymetrix CEL files were processed with the MAS5 algorithm. Then, for each probe set that corresponds to the same gene, only the probe set with the highest median value across all samples was kept. The log2 expression values were further normalized by subtracting the median expression across samples and dividing by the median absolute deviation (MAD). This process was repeated for all genes to correct for the difference in values caused by varying probe affinities. Median and MAD were used instead of mean and standard deviation as they are less prone to the effect of outliers. Following normalization, the bottom 20% quantile values across all genes were recorded for each patient and labeled as under-expressed. The resulting classification of GRM1 gene into under (GRM1-) and normal expression (GRM1+) was compared to corresponding time to distant recurrence events using Kaplan-Meier curves and Cox regression.

### Statistical Analysis

Permutation tests were performed to determine the statistical significance of differences in mean age at diagnosis between different genotype groups (e.g., wild-type homozygote, heterozygote, variant homozygote) using dominant and recessive models. This permutation test was chosen because it is non-parametric, with the assumption that all genotype groups, or categories, are equivalent and making no assumptions about the age of diagnosis distribution. Fisher’s exact test was used to determine the statistical significance of the association between categorical values for each genotype group. The odds ratio and 95% confidence interval were then computed using a Bayesian estimate for the odds ratio posterior distribution. A *P* value of less than 0.05 was used to indicate statistical significance and all statistical tests were two-sided. Two-by-two contingency tables were used to analyze correlations between the different molecular features that were examined in analysis of the tissue microarrays. Odds ratios with 95% confidence intervals were calculated and Fisher’s exact test was used to calculate a two-tailed p-value. For demographic characteristics, a chi-square test for independence was performed using the likelihood ratio test. Analyses included those cases for which all data points were available for that case. Analysis of changes in GRM1 expression after hormone and drug manipulation of cells was based on the analysis of variance (ANOVA) model together with the Duncan's Multiple Range Test for comparing all pairs of means. Comparisons to 17β-estradiol (E2) treatment were performed using Dunnett’s multiplicity adjustments. For cell proliferation studies, two-tailed t test was used to calculate the *P* value between control and doxycycline-treated cells where a *P* value of less than 0.05 was considered statistically significant.

## Results

### Demographics of the Breast Cancer Cohort

Genetic association studies were performed in a cohort of 1,028 consecutively-enrolled patients with breast cancer. Demographics and tumor characteristics for this cohort are depicted in Table 1. The data show that the majority of women were Caucasian (77.5%) and the majority of their cancers were ductal in origin (84.1%). The average age at diagnosis was 52 years, with patients ranging from 19-89 years of age. At the time of diagnosis, the majority of breast cancers were early stage (0-II). Seventy-one percent of the breast cancers were ER positive (ER+) and 56.5% were PR positive (PR+).

**Table 1:** Demographics for study participants by GRM1 locus.

Case Attribute	rs6923492				rs362962			
	CC, n (%)	TC, n (%)	TT, n (%)	in HWE	CC, n (%)	TC, n (%)	TT, n (%)	in HWE
**Ethnicity**								
African American	33 (51.6)	26 (40.6)	5 (7.8)	Yes	31 (48.4)	21 (32.8)	11 (17.2)	No
Asian	24 (33.8)	32 (45.1)	15 (21.1)	No	4 (5.6)	25 (35.2)	42 (59.2)	No
Caucasian	214 (26.9)	425 (53.5)	155 (19.5)	No	62 (7.8)	304 (38.3)	428 (53.9)	Yes
Hispanic	26 (38.8)	34 (50.8)	7 (10.5)	No	9 (13.4)	33 (49.3)	25 (37.3)	No
Other	10	15	4	n/a	2	4	12	n/a
**Mean Age at Diagnosis, years**	52							
**Median Age at Diagnosis (range)**	51 (19-89)							
	**rs6923492**				**rs362962**			
**Tumor Attribute**	**CC, n (%)**	**TC, n (%)**	**TT, n (%)**	**p- value**	**CC, n (%)**	**TC, n (%)**	**TT, n (%)**	**p- value**
Histologic Subtype								
Ductal	253	462	141		97	327	441	
Lobular^a^	33	41	19		7	43	44	
Mixed ductal/lobular	3	8	1		0	6	6	
Other^b^	16	15	6		4	10	21	
Unknown	9	6	2		1	6	9	
				0.2018				0.4626
**Stage at diagnosis**								
0	29	49	19		12	41	44	
I	112	185	71		31	140	197	
II	102	186	56		37	133	77	
III	38	59	22		16	39	64	
IV	9	25	13		6	22	19	
				0.6549				<0.0001
**Estrogen Receptor (ER)**								
Positive	214	376	140		67	278	385	
Negative	72	130	40		34	94	113	
				0.6415				0.0732
**Progesterone Receptor (PR)**								
Positive	164	297	117		49	222	320	
Negative	109	184	59		47	139	164	
				0.3894				0.0177
**Her2 status^c^**								
Positive	50	81	34		15	62	87	
Negative	176	307	110		67	228	297	
Equivocal	2	11	0		0	6	7	
				0.0814				0.4767

a excludes lobular carcinoma in situ; ^b^other includes colloid, medullary, apocrine, metaplastic, inflammatory cancers. ^c^positive defined as either FISH ≥2.1 or 3+ by immunohistochemistry. Her2 defined as equivocal for FISH 1.9-2.09 or IHC 2+ in the absence of FISH. Cases for which genotype or phenotype were not available are excluded.

### Association between GRM1 SNPs and Breast Cancer Molecular Subtypes

Three GRM1 SNPs, rs6923492, rs362962, and rs1125462, were evaluated in the breast cancer cohort for possible associations with breast cancer phenotypes. At the rs6923492 locus, the CC genotype occurred at frequencies of 51.6% and 26.9% in African Americans and Caucasians, respectively. Similarly, rs362962 showed race-specific genotype frequencies where the CC genotype was prevalent at 48.4% and 7.8% in African American and Caucasian cases, respectively. These genotype distributions reflect reported ancestral alleles. Genotype distributions for Asians (rs6923492 CC: 33.8%; rs362962 CC: 5.6%) and Hispanics (rs6923492 CC: 38.8%; rs362962 CC: 13.4%) were more similar to Caucasians. Deviations from HWE occurred for rs6923492 in Caucasian and for rs362962 in African Americans cases only.

There were no genotype-specific differences in histologic subtype of breast cancer for either GRM1 locus. For rs6923492, there were no associations with stage at diagnosis, estrogen receptor (ER) status, progesterone receptor (PR) status, or Her2 status. For rs362962, there was no genotype-specific correlation with Her2 status. However, stage at diagnosis and PR status showed significant genotype associations. There was enrichment for stage I disease in TT carriers as compared to nearly equal distribution of stage I and stage II in rs362962-C allele carriers. The rs362962-T allele was more likely to be PR+ (p = 0.018). A similar distribution was observed for rs362962-T allele and ER positivity, but this did not reach statistical significance (p = 0.073).

### Gene Associations and Breast Cancer Phenotypes

The cumulative incidence of ductal carcinomas by age of diagnosis was evaluated for associations with GRM1 genotypes. Because of variable race-specific allele frequencies and tumor heterogeneity, gene association analyses were limited to Caucasians with ductal carcinomas. Analyses were then performed separately for ER+/PR+ and ER−/PR- ductal carcinomas given the known heterogeneous biologic behavior and demographics associated with these subtypes.

**Figure 1:**
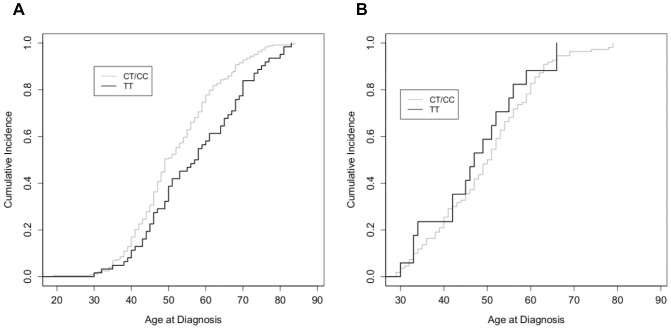
Cumulative incidence of breast cancer as a function of age of diagnosis for rs6923492 genotypes. Fraction of individuals diagnosed with breast cancer as a function of age, in a cohort of Caucasian women diagnosed with (A) ER+/PR+ ductal breast cancer and (B) ER−/PR- ductal breast cancer as a function of rs6923492 genotype. Data were analyzed as TT (black) versus C allele (grey).

For GRM1 rs6923492, ER+/PR+ ductal carcinomas in TT carriers occurred at a later age as compared to either TC or CC carriers, corresponding to the right shift in the curve (Fig. 1A). Due to the similarity between TC and CC genotypes, analysis of age at diagnosis was performed for the C allele vs. TT resulting in a significant difference between the curves (p = 0.0076). The later age at diagnosis was 4.9 years in TT carriers. There was no difference in age at diagnosis for ER−/PR- ductal carcinomas (Fig. 1B).

**Figure 2:**
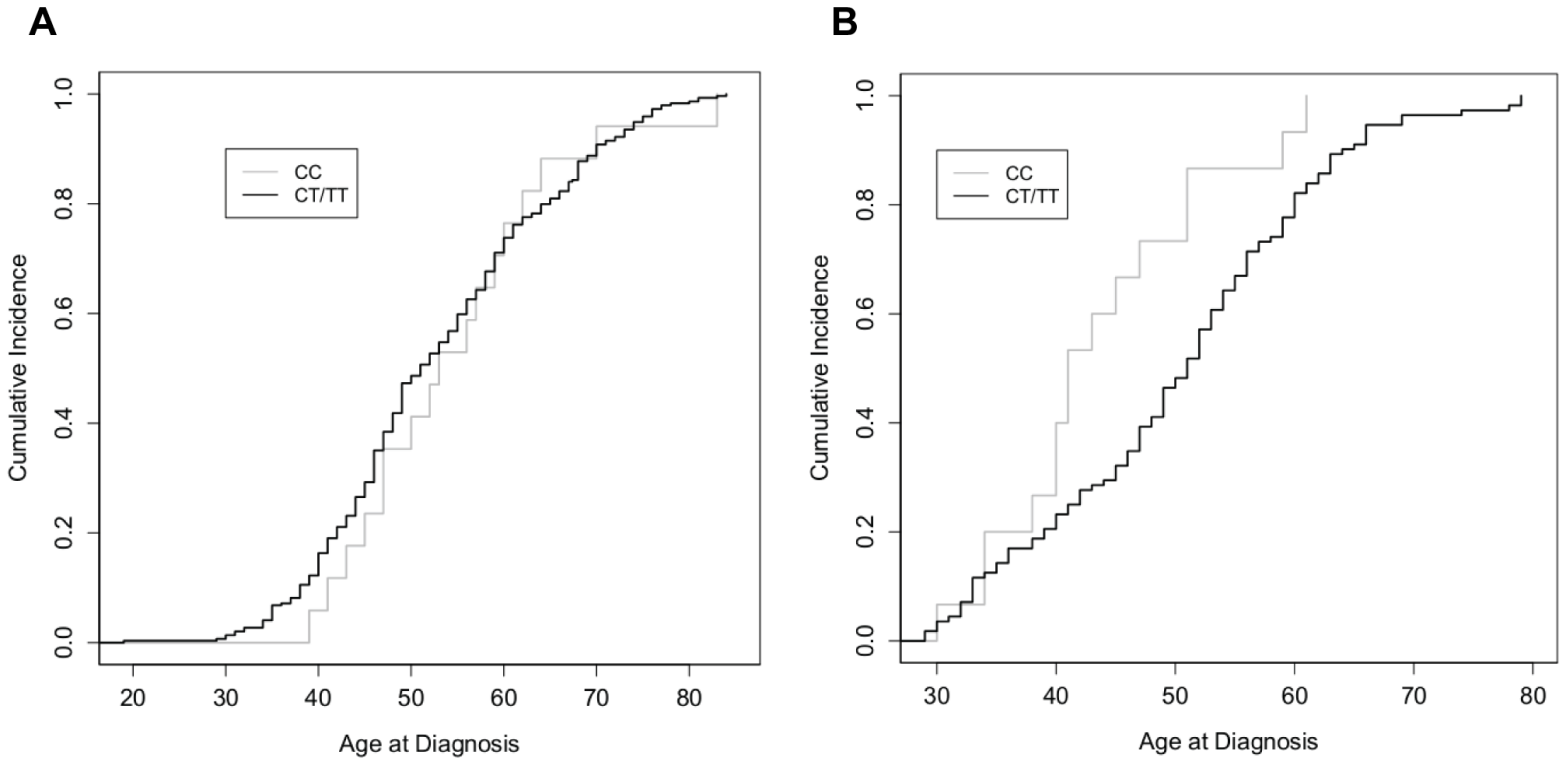
Cumulative incidence of breast cancer as a function of age of diagnosis for rs362962 genotypes. Fraction of individuals diagnosed with breast cancer as a function of age, in a cohort of Caucasian women diagnosed with (A) ER+/PR+ ductal breast cancer and (B) ER−/PR- ductal breast cancer as a function of rs362962 genotype. Data were analyzed as CC (grey) versus T allele (black).

In contrast to rs6923492, GRM1 rs362962 demonstrated different patterns of associations based on hormone receptor status (Fig. 2). For ER−/PR- ductal carcinomas, disease in CC carriers occurred 4.9 years and 6.7 years earlier than TT and CT carriers, respectively (p = 0.049; p = 0.027). Since the heterozygotes were similar to TT carriers, analysis was performed for T allele vs. CC curves (Fig. 2B, p = 0.029). For this combined analysis, CC carriers were diagnosed, on average, 5.7 years earlier than in T allele carriers. There was no difference in age at diagnosis for ER+/PR+ ductal carcinomas (Fig. 2A).

There was a significant correlation between the CC genotype of rs362962 with the development of hormone receptor negative breast cancer. CC carriers had a higher probability of having ER- breast cancer (odds ratio [O.R.] 1.73; 95% confidence interval [CI], 1.09-2.75; p = 0.019) or PR- breast cancer (O.R. 1.87; 95% CI, 1.20-2.91; P = 0.005) than those carrying the TT genotype. Conversely, TT carriers were more likely to have ER+ or PR+ breast cancers.

### Effect of GRM1 Expression on ER+ Breast Cancer Cell Proliferation

**Figure 3:**
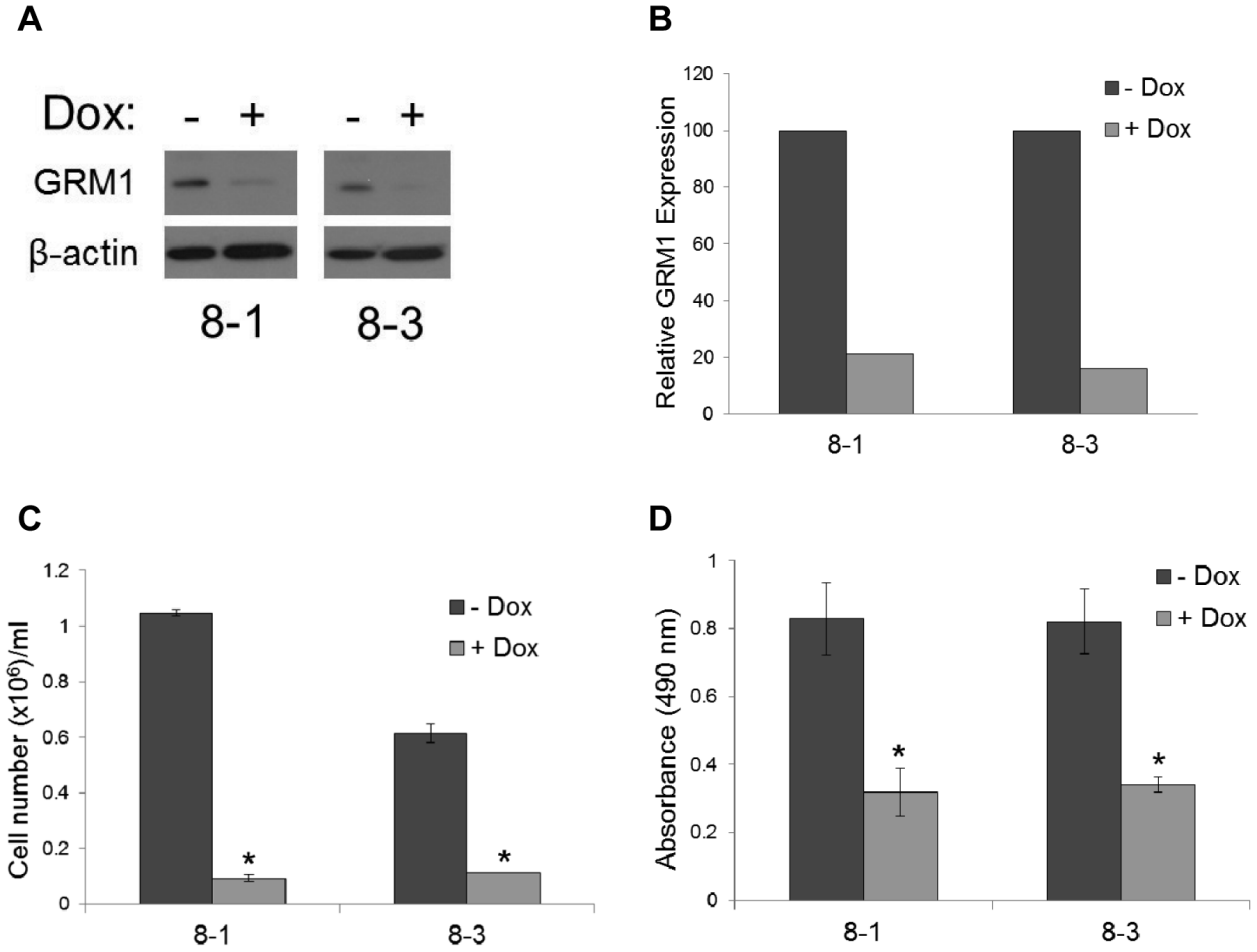
Reduced GRM1 expression alters proliferation of ER+ breast cancer cells. (A) Western blot of inducible MCF7 siGRM1 clones (8-1 and 8-3) +/− doxycycline (Dox) for ten days. (B) Quantitation of western blots normalizing GRM1 expression to β-actin indicates GRM1 knockdown to 21% and 16% of control in 8-1 and 8-3 clones, respectively. (C) Cell number is significantly reduced in cells with GRM1 knockdown as compared to control cells. (D) GRM1 knockdown decreases cell proliferation as compared to control cells as measured by MTS assay. In (C) and (D), data represent the mean value of triplicate experiments +/− SD. *P<0.05.

As one potential cause of earlier age at diagnosis of breast cancer may reflect faster growing tumors, the effect of GRM1 expression on breast cancer cell growth was evaluated. MCF7 cells were stably transfected with a doxycycline-inducible siGRM1 vector that results in conditional knockdown of GRM1 in the presence of doxycycline. MCF7 siGRM1 cells grown in the absence of doxycycline served as an isogenic control cell. Western blot analysis of GRM1 confirmed knockdown of expression (Fig. 3A and B). Upon knockdown of GRM1 in two independent stable cell lines, cell number was significantly reduced indicating that reduced GRM1 expression inhibits cell proliferation (Fig. 3C). Additionally, measurement of cell proliferation by MTS assay produced similar results suggesting that GRM1 may be an important regulator of breast cancer cell growth (Fig. 3D).

### Correlation of GRM1 Expression and Molecular Features of Breast Cancer from a Breast Tissue Microarray

**Figure 4:**
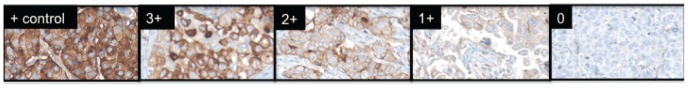
Immunohistochemical staining for GRM1 on tissue microarrays of human breast tissue. Representative examples of the IHC scoring system using breast cancer tissues are shown. Melanoma (left panel) was utilized as a positive control and for antibody optimization. Scoring was based on intensity of membrane staining: 0; 1+; 2+; 3+.

Given the correlations between GRM1 SNP genotypes and hormone receptor status, GRM1 expression was evaluated in a set of breast tissue microarrays for correlation with molecular features of breast cancer, e.g. receptor status. Figure 4 shows the staining patterns for scoring of GRM1 by IHC. The percentage of GRM1 positivity was higher in breast cancers (73%) as compared with normal breast tissue (15%, p<0.0001) (Table 2). Furthermore, ER+ breast cancer was also significantly more likely to be GRM1+ (p<0.002) where 83% of ER+ tumors were also GRM1+ but only 66% of ER- tumors were GRM1+. Although no significant correlation was found for PR status, breast cancers that were both ER+/PR+, were again more likely to be GRM1+, compared to cancers that were ER−/PR- (O.R. 2.3, 95% CI [1.3-4.5], p = 0.009).

**Table 2:** Evaluation of immunohistochemical staining of GRM1 in breast cancer and correlation with molecular features from a breast tissue microarray.

		GRM1 Expression		
Feature	Total, n	Positive,n (%)	Negative,n (%)	p-value
**Histology**				
Normal	71	11 (15)	60 (85)	
Cancer	324	236 (73)	88 (27)	<0.0001
**ER Status**				
Positive	138	115 (83)	23 (17)	
Negative	144	95 (66)	48 (34)	<0.002
**PR Status**				
Positive	125	99 (79)	26 (21)	
Negative	157	111 (71)	46 (29)	0.13
**ER/PR Status**				
Positive/Positive	99	81 (82)	18 (18)	
Negative/Negative	118	77 (65)	41 (35)	<0.01

### Effect of Hormonal Manipulation on GRM1 Expression

**Figure 5:**
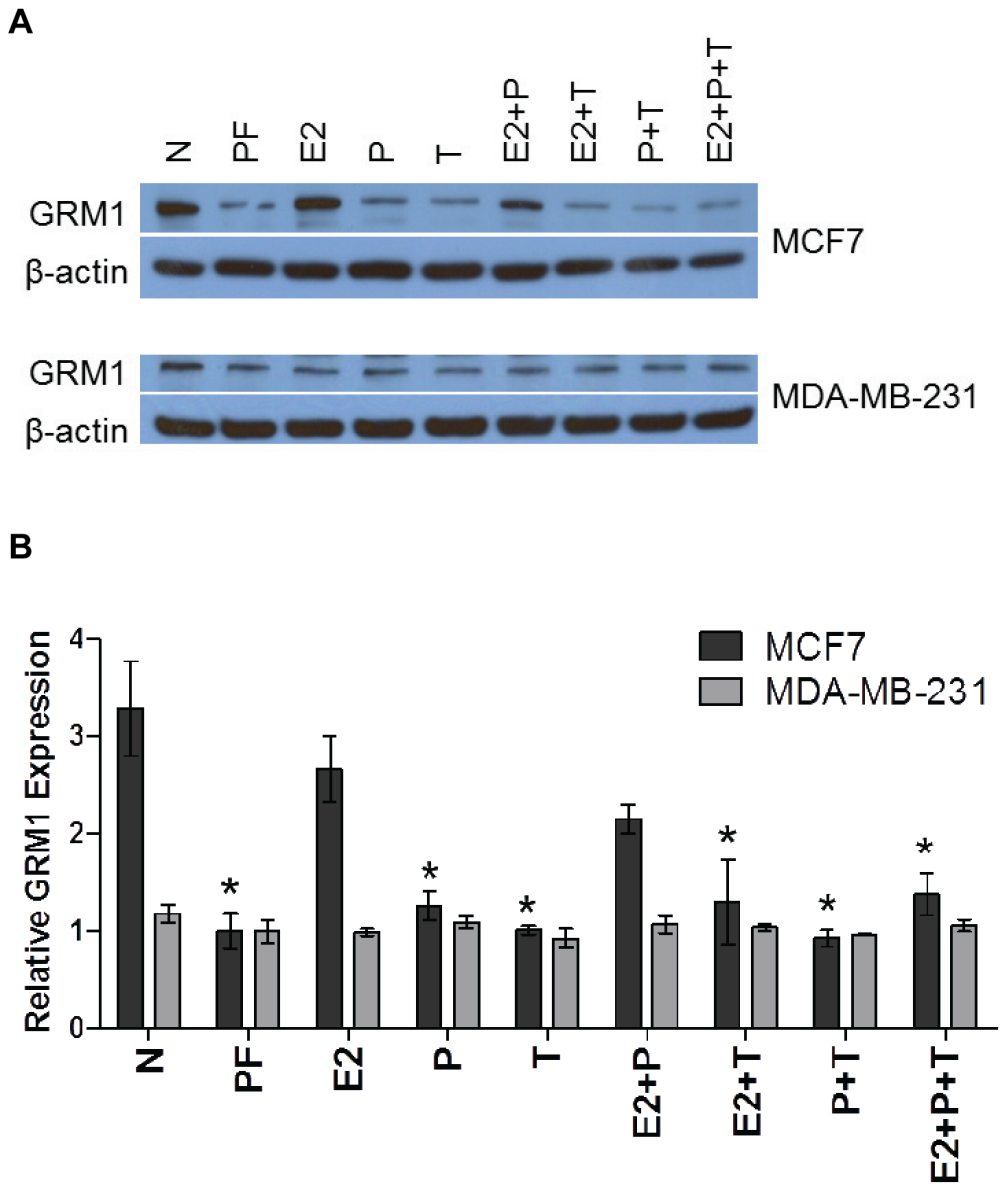
17β-estradiol induction of GRM1 protein expression in ER+ breast cancer cells is abrogated by 4-hydroxytamoxifen. MCF7 (ER+/PR+) and MDA-MB-231 (ER−/PR-) breast cancer cells were treated with 17β-estradiol (E2), progesterone (P), 4-hydroxytamoxifen (T), or a combination of hormonal agents for a total of 48 h. (A) Western blot analysis uses β-actin for protein loading control. N = normal growth medium, PF = phenol-free, charcoal-stripped growth medium. (B) Quantitation of western blots from three independent experiments. Data represent the mean value of triplicate experiments +/− SE. * *P*<0.05 compared to E2 treatment.

To determine if estrogen or progesterone affects GRM1 expression *in vitro,* western blot analysis was used to examine GRM1 protein expression after hormone manipulation in the following breast cancer cell lines: MCF7 (ER+/PR+) and MDA-MB-231 (ER−/PR-). Cells were treated with 17β-estradiol, progesterone or the selective estrogen receptor modulator, 4-hydroxytamoxifen. Figure 5 shows the effect of these treatments on GRM1 protein expression in the MCF7 and MDA-MB-231 cell lines.

In MCF7 cells, GRM1 expression increased after treatment with 17β-estradiol and the combination of 17β-estradiol and progesterone, as compared to phenol-free (PF-DMEM) conditions (Fig. 5). Specifically, in MCF7 cells, GRM1 expression increased 2.7 fold after 17β-estradiol treatment and 2.1-fold after 17β-estradiol plus progesterone treatment as compared to PF-DMEM conditions. Cells treated in PF-DMEM remove estrogen and estrogen-like compounds that are normally present in untreated media and therefore would be predicted to be more similar to results in tamoxifen-treated cells. Co-treatment of MCF7 cells with tamoxifen blocked the increase in GRM1 expression with 17β-estradiol and with the combination of 17β-estradiol and progesterone. As expected, no changes in GRM1 expression were observed in the hormone receptor negative MDA-MB-231 breast cancer cell line with any of the hormonal treatments.

### Association between GRM1 Expression and Breast Cancer Recurrence

**Figure 6:**
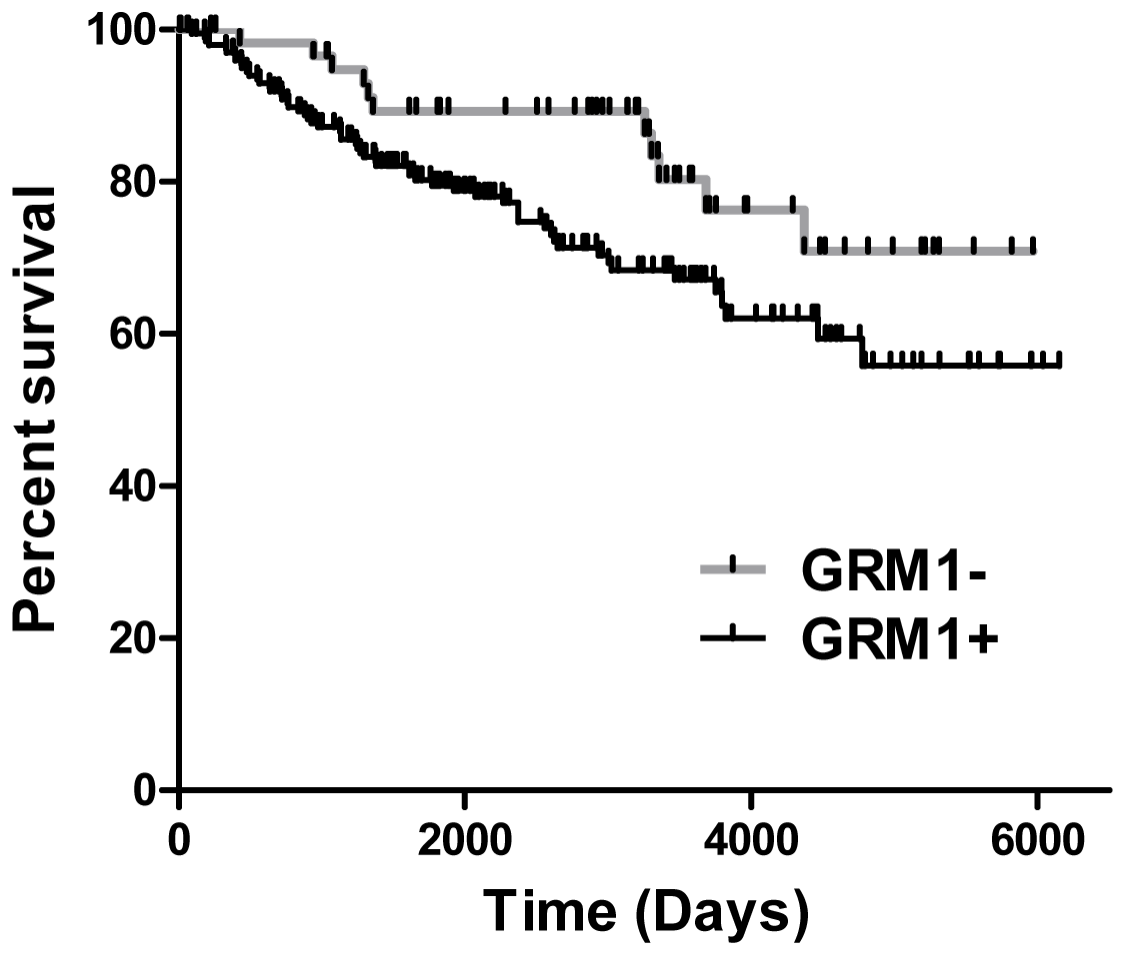
DMFS as a function of GRM1 mRNA expression in breast cancer patients treated with tamoxifen. Kaplan-Meier curve describing distant metastasis free survival (DMFS) of 268 patients, with ER-positive breast cancer, treated with tamoxifen as a function of GRM1 expression. Curve is generated from reanalysis of data from Loi *et al.* [32]. 68 patients are in the low GRM1 expressor group (GRM1-); 200 patients are represented in the high GRM1 expressing group (GRM1+).

Associations between GRM1 expression in breast tumors and breast cancer outcomes have not been previously reported. Therefore, existing data in public databases were mined to evaluate this association. One such dataset is that of Loi *et al.* [32], who studied gene expression patterns of primary ER+ breast tumors and response to tamoxifen. Gene expression data was re-analyzed for distant metastasis-free survival (DMFS) in 268 tamoxifen-treated patients as a function of GRM1 expression (Fig. 6). Patients were stratified into 62 GRM1- and 206 GRM1+ cases based on “low” and “high” expression values of the GRM1 gene (as defined in Methods). This analysis revealed that low GRM1 expression associated with longer DMFS as compared to higher GRM1 expression. According to the log-rank test, DMFS was significantly different between low and high expression of GRM1 (Hazard Ratio 0.57, CI [0.33-0.97], p = 0.0380).

## Discussion

A genetic and molecular study of metabotropic glutamate receptor 1 (GRM1) in human breast cancer was conducted. Three GRM1 SNPs were evaluated for associations with breast cancer clinicopathologic variables. To reduce cohort heterogeneity and to eliminate known clinical biases in young-onset disease, cases were stratified by molecular subtype (*i.e.* ER/PR status), histologic subtype (*i.e.* ductal), and race. The GRM1 rs6923492 locus significantly associated with an age at diagnosis phenotype for estrogen receptor positive/progesterone receptor positive disease, while the rs362962 locus association was strongest for estrogen receptor negative/progesterone receptor negative disease for Caucasian ductal carcinomas. In addition, the TT genotype for rs362962 was associated with higher risk of estrogen receptor positive or progesterone receptor positive disease. Based on the observed associations between GRM1 SNPs with receptor status, GRM1 protein expression was evaluated in human breast tissue where it was found that breast tumors had a higher probability of expressing GRM1 as compared to normal tissue and that GRM1 positivity was most highly correlated with estrogen receptor positivity. *In vitro*, GRM1 protein expression was subsequently observed to increase with estrogen or estrogen and progesterone treatment that was inhibited by tamoxifen treatment. Furthermore, in primary estrogen receptor positive human breast tumors with high GRM1 expression, there was shorter distant metastasis-free survival with tamoxifen treatment. These data are suggestive of a functional role for GRM1 in the setting of active estrogen signaling.

Analysis of data from the GRM1 gene association study revealed deviations from Hardy-Weinberg equilibrium (HWE) for rs6923492 in the Caucasian and rs362962 in the African American populations. The test for HWE assumes the absence of migration, mutation, natural selection, assortative mating and an infinite size in population. For GRM1 rs6923492, the apparent deviation of HWE could be attributable to a selection bias towards the heterozygote genotype, which was more evident in Caucasians than other populations, true disease association, or population stratification. Assay failure was excluded by duplicative genotyping and direct sequencing of a number of randomly selected samples. Deviation from HWE for rs362962 in the African American cases was more likely due to small sample size and/or admixture.

As with prior studies with this cohort, associations were observed for GRM1 SNPs that were highly dependent on receptor status of the breast cancer [33]-[37]. This is consistent with numerous genome wide association, pathway-focused, and candidate gene studies that have found receptor status-specific genetic associations for risk and for breast cancer outcomes in both BRCA mutation-associated and sporadic breast cancers [38]-[48]. Given the diverse gene expression patterns and biologic behavior of individual breast cancer subtypes, distinct genetic background might be anticipated as one possible mechanism giving rise to the observed heterogeneity of tumor types.

Although the gene association studies correlated with clear clinical phenotypes, the mechanism of functionality of either GRM1 rs6923492 or rs362962 remains undefined. However, we predict that because GRM1 SNP rs6923492 results in a proline to serine amino acid substitution, this could potentially affect functionality either through altered expression or through altered function. The former is unlikely as EBV-transformed lymphoblastoid cell lines representative of each genotype express equal amounts of GRM1 protein (data not shown). An amino acid change of this type could result in altered protein backbone flexibility leading to altered protein structure and hence, function. In addition to this amino acid being located near the carboxy terminus of GRM1, it is also near the intracellular portion of this transmembrane protein [49]. Functionality of GRM1 occurs through GRM1-intracellular protein interactions, e.g. G proteins. Therefore, altered structure could impact these interactions and efficacy of transducing downstream signals as a result of GRM1 activation.

As we hypothesized that the mechanism of functionality of GRM1 rs6923492 is altered function through protein interactions, we performed *in silico* exploration of potential phosphorylation targets using a web-based phospho-motif finder [50]. Our analysis reveals that the GRM1 rs6923492 proline to serine change potentially affects binding to target kinase proteins and its phosphorylation (Table 3). The serine, but not the proline residue at amino acid position 993, predicts for interaction of GRM1 with the following proteins based on substrate-specific motifs: G-protein coupled receptor kinase 1, DNA-dependent protein kinase, and casein kinase I and II. G-protein coupled receptor kinase 1 (GRK1), a serine-threonine kinase, is a member of the GRK family of receptor kinases which function to modulate GPCR signaling through binding of agonist-activated GPCRs. As a result of GRK binding, β-arrestin is recruited and sterically inhibits coupling between GPCR and its G-protein, leading to diminished GPCR signaling. This mechanism has been shown to occur with GRM1 in other tissue types [51]. In the context of the GRM1 SNP of interest, the serine residue (TT genotype), but not the proline residue, may create a putative phosphorylation site for GRK that may lead to inhibition of GRM1 signaling. As a GPCR, GRM1 interaction with GRK1 might be predicted.

**Table 3:** *In silico* analysis of potential phosphorylation of GRM1 amino acid residues as a function of the nonsynonymous SNP rs6923492.

Serine at amino acid 993			
Amino acid position in GRM1	Sequence in GRM1	Corresponding motif	Features of motif
983-985	PSA	P[pS/pT]X	DNA dependent protein kinase substrate motif
983-988	PSAATT	X[pS/pT]XXX[A/P/S/T]	G protein-coupled receptor kinase I substrate motif
984-987	SAAT	[pS/pT]XX[S/T]	Casein kinase I substrate motif
984-987	SAAT	pSXX[E/pS*/pT*]	Casein kinase II substrate motif
984-988	SAATT	pSXXX[pS/pT]	MAPKAPK2 kinase substrate motif
987-989	TTP	X[pS/pT]P	GSK-3, ERK1, ERK2, CDK5 substrate motif
992-994^a^	PSH	P[pS/pT]X	DNA dependent protein kinase substrate motif
992-997^a^	PSHLTA	X[pS/pT]XXX[A/P/S/T]	G protein-coupled receptor kinase I substrate motif
993-996^a^	SHLT	[pS/pT]XX[S/T]	Casein kinase I substrate motif
993-996^a^	SHLT	pSXX[E/pS*/pT*]	Casein kinase II substrate motif
**Proline at amino acid 993**			
**Amino acid position in GRM1**	**Sequence in GRM1**	**Corresponding motif**	**Features of motif**
983-985	PSA	P[pS/pT]X	DNA dependent protein kinase substrate motif
983-988	PSAATT	X[pS/pT]XXX[A/P/S/T]	G protein-coupled receptor kinase I substrate motif
984-987	SAAT	[pS/pT]XX[S/T]	Casein kinase I substrate motif
984-987	SAAT	pSXX[E/pS*/pT*]	Casein kinase II substrate motif
984-988	SAATT	pSXXX[pS/pT]	MAPKAPK2 kinase substrate motif
987-989	TTP	X[pS/pT]P	GSK-3, ERK1, ERK2, CDK5 substrate motif
995-1000	LTAEET	X[pS/pT]XXX[A/P/S/T]	G protein-coupled receptor kinase I substrate motif

a denotes novel putative binding of kinases and phosphorylation of amino acid residues in GRM1 when serine was present at residue 993, but not proline at the same position.

There have been no previous studies investigating GRM1 interaction with DNA-dependent protein kinase (DNA-PK), a repair protein that participates in non-homologous end joining. DNA-PK has been implicated in the processing of oxidation-induced DNA damage. In neostriatal neurons, GRM1 activation by binding of a group I mGluR agonist results in activation of casein kinase 1 (CK1), a highly conserved serine-threonine kinase which was recently implicated as a tumor suppressor in melanoma [52], [53]. Furthermore, 19 nonsynonymous SNPs in CK1 identified in breast cancer patients were shown to have a significant association with loss of heterozygosity and decreased staining of CK1 in tumor tissues [54]. Given the observed association between activation of GRM1 with that of CK1, it is postulated that the role of CK1 as a tumor suppressor may be mediated by a negative feedback loop with GRM1. Although four putative kinases were identified through our analysis, binding of kinases and phosphorylation of amino acid residues in GRM1 can be modulated by other amino acid residues in the vicinity. Therefore, it is possible that other kinases may be involved in the hypothesized discrepant function between proline and serine isoforms of GRM1 beyond those presented in Table 3. Unlike rs6923492, potential mechanisms for functionality of the intronic SNP rs362962 remain unclear as evaluation for potential altered miR or transcription factor binding sites, splice sites, GRM1 expression, or the same for linked SNPs was unrevealing.

Beyond SNP functionality, altered GRM1 expression displayed a phenotype in breast cancer cell behavior. Induced knockdown of GRM1 in an ER+ breast cancer cell line correlated with reduced cell proliferation. This is consistent with data by Speyer *et al.* [31] where they recently reported a role for GRM1 in the pro-proliferative phenotype of triple negative breast cancer cells. This phenotype could be inhibited by treatment with a GRM1 inhibitor or with shRNA resulting in increased apoptosis both *in vitro* and in a mouse xenograft model. However, Speyer *et al.* [31] did not evaluate GRM1 in the setting of active estrogen signaling or with human patients. Positive associations seen in analysis of distant metastasis free survival as a function of GRM1 expression in tamoxifen-treated patients as well as the hormone receptor-dependent associations seen for GRM1 SNPs implicate a potential role between hormone signaling and the GRM1 pathway. TMA analysis of GRM1 expression in breast tumors further confirmed the correlation between hormone receptor status and GRM1 expression. As no further changes in GRM1 expression were observed with the addition of progesterone to breast cancer cells *in vitro*, estrogen appears to have the greatest effect on GRM1 expression. Although high GRM1 expression in primary tumors correlated with shorter distant metastasis-free survival, data may be limited by small sample size and should be validated in a larger cohort. Distant metastasis free survival was used as a surrogate endpoint for overall survival, but it may be more appropriate to use overall survival due to late recurrences and lengthy survivals even in the setting of metastatic disease. However, its use as a surrogate may be justifiable given that recurrence of disease at a distant site would be expected to contribute to mortality in this population since metastatic disease is not curable in this setting [55].

The fact that GRM1 expression is prominent in malignant breast tissue as compared to normal breast tissue suggests that altered GRM1 expression occurs in the development of breast cancer. Our observations in breast tissue are consistent with a recent report of upregulated GRM1 expression in malignant as compared to normal prostate tissue [26]. The pro-proliferative, pro-growth, and anti-apoptotic signals occurring as a result of GRM1 over-expression and activation would provide a survival advantage for GRM1-expressing breast cancer cells. In fact, the positive correlation between GRM1 expression and cell proliferation was confirmed in this study. Activation of GRM1 leads to increased MAPK and AKT activities where both are known to contribute to resistance to breast cancer endocrine therapies including tamoxifen [27], [28], [30], [56]-[59].

In summary, it was found that GRM1 expression and two polymorphic variants associate with breast cancer phenotypes. Because of the associations with estrogen receptor status, this data implicates GRM1 as having a functional role in ER+ breast cancer where its expression associates with response to tamoxifen. Further studies are needed to determine how GRM1 may affect survival and how variants in GRM1 become mechanistically functional.

## References

[1] Siegel R, Naishadham D, Jemal A (2013) Cancer statistics, 2013. CA Cancer J Clin 63: 11–30. 23335087 10.3322/caac.21166

[2] Antoniou A, Pharoah PD, Narod S, Risch HA, Eyfjord JE, et al (2003) Average risks of breast and ovarian cancer associated with BRCA1 or BRCA2 mutations detected in case series unselected for family history: A combined analysis of 22 studies. Am J Hum Genet 72: 1117–1130. 12677558 10.1086/375033 PMC1180265

[3] Hirshfield KM, Rebbeck TR, Levine A. (2010) Germline mutations and polymorphisms in the origins of cancers in women. J Oncology 2010: 297671 [doi:10.1155/2010/297671]. 10.1155/2010/297671 PMC2810468 20111735

[4] Wu PE, Shen CY (2011) 'Hide-then-hit' to explain the importance of genotypic polymorphism of DNA repair genes in determining susceptibility to cancer. J Mol Cell Biol 3: 59–65. 21278453 10.1093/jmcb/mjq054

[5] Bugano DD, Conforti-Froes N, Yamaguchi NH, Baracat EC (2008) Genetic polymorphisms, the metabolism of estrogens and breast cancer: a review. Eur J Gynaecol Oncol 29: 313–20. 18714561

[6] Wagner K, Hemminki K, Försti A (2007) The GH1/IGF-1 axis polymorphisms and their impact on breast cancer development. Breast Cancer Res Treat 104: 233–48. 17082888 10.1007/s10549-006-9411-9

[7] Zheng W (2009) Genetic polymorphisms in the transforming growth factor-beta signaling pathways and breast cancer risk and survival. Methods Mol Biol 472: 265–77. 19107437 10.1007/978-1-60327-492-0_11

[8] Peng S, Lü B, Ruan W, Zhu Y, Sheng H, Lai M (2011) Breast cancer genome-wide association studies: there is strength in numbers. Breast Cancer Res Treat 127: 309–24. 21445572 10.1007/s10549-011-1459-5

[9] Monnerat C, Chompret A, Kannengiesser C, Avril MF, Janin N, et al (2007) BRCA1, BRCA2, TP53, and CDKN2A germline mutations in patients with breast cancer and cutaneous melanoma. Familial Cancer 6: 453–461. 17624602 10.1007/s10689-007-9143-y

[10] Ortiz P, Vanaclocha F, López-Bran E, Esquivias JI, López-Estebaranz JL, et al (2007) Genetic analysis of the GRM1 gene in human melanoma susceptibility. Eur J Hum Genet 15: 1176–82. 17609672 10.1038/sj.ejhg.5201887

[11] Monaghan DT, Bridges RJ, Cotman CW (1989) The excitatory amino acid receptors: their classes, pharmacology, and distinct properties in the function of the central nervous system. Annu Rev Pharmacol Toxicol 29: 365–402. 2543272 10.1146/annurev.pa.29.040189.002053

[12] Hollmann M, Heinemann S (1994) Cloned glutamate receptors. Annu Rev Neurosci 17: 31–108. 8210177 10.1146/annurev.ne.17.030194.000335

[13] Belcheva M, Coscia CJ (2002) Diversity of G protein-coupled receptor signaling pathways to ERK/MAP kinase. Neurosignals 11: 34–44. 11943881 10.1159/000057320 PMC2581518

[14] Hermans E, Challis RA (2001) Structural, signaling and regulatory properties of the group I metabotropic glutamate receptors: prototypic family C G-protein-coupled receptors. Biochem J 359(Pt 3): 465–84. 10.1042/0264-6021:3590465 PMC1222168 11672421

[15] Condorelli DF, Dell'Albani P, Corsaro M, Giuffrida R, Caruso A, et al (1997) Metabotropic glutamate receptor expression in cultured rat astrocytes and human gliomas. Neurochem Res 22: 1127–33. 9251103 10.1023/a:1027317319166

[16] Pollock PM, Cohen-Solal K, Sood R, Namkoong J, Martino JJ, et al (2003) Melanoma mouse model implicates metabotropic glutamate receptor signaling in melanocytic neoplasia. Nat Genet 34: 108–12. 12704387 10.1038/ng1148

[17] Shin SS, Martino JJ, Chen S (2008) Metabotropic receptors and cellular transformation. Neuropharmacology 55: 396–402. 18554669 10.1016/j.neuropharm.2008.04.021 PMC2605077

[18] Shin SS, Namkoong J, Wall BA, Gleason R, Lee HJ, Chen S (2008) Oncogenic activities of metabotropic glutamate receptor 1 (Grm1) in melanocyte transformation. Pigment Cell Melanoma Res 21: 368–378. 18435704 10.1111/j.1755-148X.2008.00452.x PMC2854004

[19] Martino JJ, Wall BA, Mastrantoni E, Wilimczyk BJ, La Cava SN, et al.. (2012) Metabotropic glutamate receptor (Grm1) is an oncogene in epithelial cells. Oncogene [doi: 10.1038/onc.2012.471]. 10.1038/onc.2012.471 PMC3910169 23085756

[20] Marin YE, Namkoong J, Shin SS, Raines J, Degenhardt K, et al.. (2005) Grm5 expression is not required for the oncogenic role of Grm1 in melanocytes. Neuropharmacology (Suppl 1): 70–9. 10.1016/j.neuropharm.2005.05.018 16040064

[21] Le MN, Chan JL, Rosenberg SA, Nabatian AS, Merrigan KT, et al (2010) The glutamate release inhibitor Riluzole decreases migration, invasion, and proliferation of melanoma cells. J Invest Dermatol 130: 2240–9. 20505744 10.1038/jid.2010.126 PMC4004181

[22] Wangari-Talbot J, Wall BA, Goydos JS, Chen S (2012) Functional effects of GRM1 suppression in human melanoma cells. Mol Cancer Res 10: 1440–50. 22798429 10.1158/1541-7786.MCR-12-0158 PMC3501593

[23] Lee HJ, Wall BA, Wangari-Talbot J, Shin SS, Rosenberg S, et al (2011) Glutamatergic pathway targeting in melanoma: single-agent and combinatorial therapies. Clin Cancer Res 17: 7080–92. 21844014 10.1158/1078-0432.CCR-11-0098 PMC3218300

[24] Namkoong J, Shin SS, Lee HJ, Marin YE, Wall BA, et al (2007) Metabotropic glutamate receptor 1 and glutamate signaling in human melanoma, Cancer Res. 67: 2298–2305. 10.1158/0008-5472.CAN-06-3665 17332361

[25] Seidlitz EP, Sharma MK, Saikali Z, Ghert M, Singh G (2009) Cancer cell lines release glutamate into the extracellular environment. Clin Exp Metastasis 26: 781–7. 19526315 10.1007/s10585-009-9277-4

[26] Koochekpour S, Majumdar S, Azabdaftari G, Attwood K, Scioneaux R, et al (2012) Serum Glutamate Levels Correlate with Gleason Score and Glutamate Blockade Decreases Proliferation, Migration, and Invasion and Induces Apoptosis in Prostate Cancer Cells. Clin Cancer Res 18: 5888–5901. 23072969 10.1158/1078-0432.CCR-12-1308 PMC3492499

[27] Marin YE, Namkoong J, Cohen-Solal K, Shin SS, Martino JJ, et al (2006) Stimulation of oncogenic metabotropic glutamate receptor 1 in melanoma cells activates ERK1/2 via PKCepsilon. Cellular Signaling 18: 1279–1286. 10.1016/j.cellsig.2005.10.012 16305822

[28] Shin SS, Wall BA, Goydos JS, Chen S (2010) AKT2 is a downstream target of metabotropic glutamate receptor 1 (Grm1). Pigment Cell Melanoma Res 23: 103–11. 19843246 10.1111/j.1755-148X.2009.00648.x PMC2810105

[29] Yip D, Le MN, Chan JL, Lee JH, Mehnert JA, et al (2009) A phase 0 trial of riluzole in patients with resectable stage III and IV melanoma. Clin Cancer Res 15: 3896–902. 19458050 10.1158/1078-0432.CCR-08-3303 PMC2812866

[30] Gee JM, Robertson JF, Ellis IO, Nicholson RI (2001) Phosphorylation of ERK1/2 mitogen-activated protein kinase is associated with poor response to anti-hormonal therapy and decreased patient survival in clinical breast cancer. Int J Cancer 95: 247–254. 11400118 10.1002/1097-0215(20010720)95:4<247::aid-ijc1042>3.0.co;2-s

[31] Speyer CL, Smith JS, Banda M, DeVries JA, Mekani T, Gorski DH (2012) Metabotropic glutamate receptor-1: a potential therapeutic target for the treatment of breast cancer. Breast Cancer Res Treatment 132: 565–73. 10.1007/s10549-011-1624-x PMC3898178 21681448

[32] Loi S, Haibe-Kains B, Desmedt C, Wirapati P, Lallemand F, et al (2008) Predicting prognosis using molecular profiling in estrogen receptor-positive breast cancer treated with tamoxifen. BMC Genomics 9: 239–50. 18498629 10.1186/1471-2164-9-239 PMC2423197

[33] Bond G, Hirshfield KM, Kirchhoff, Alexe G, Bond EE, et al (2006) MDM2 SNP309 accelerates tumor formation in a gender-specific and hormone-dependent manner. Cancer Res 66: 5104–10. 16707433 10.1158/0008-5472.CAN-06-0180

[34] Kulkarni D, Vazquez A, Haffty B, Bandera E, Hu W, et al (2009) A polymorphic variant in MDM4 associates with accelerated age of onset of estrogen receptor negative breast cancer. Carcinogenesis 30: 1910–5. 19762336 10.1093/carcin/bgp224 PMC3895963

[35] Mehta MS, Vazquez A, Kulkarni DA, Kerrigan JE, Atwal G, et al (2010) Polymorphic variants in TSC1 and TSC2 and their association with breast cancer phenotypes. Breast Cancer Res Treatment 125: 861–8. 10.1007/s10549-010-1062-1 PMC3876413 20658316

[36] Vazquez A, Kulkarni D, Grochola LF, Bond GL, Barnard N, et al (2011) A genetic variant in a PP2A regulatory subunit encoded by the PPP2R2B gene associates with altered breast cancer risk and recurrence. International J Cancer 128: 2335–43. 10.1002/ijc.25582 PMC3902652 20669227

[37] Haffty BG, Goyal S, Green C, Schiff D, Yang Q, et al (2011) Evaluation Of Single Nucleotide Polymorphisms (SNP) In The P53 Binding Protein Gene (TP53BP1), In Breast Cancer Patients Treated With Breast Conserving Surgery And Whole Breast Irradiation (BCS+RT). International J Radiation Oncology, Biology, Biophysics 80: 385–391. 10.1016/j.ijrobp.2010.02.005 PMC3876421 20646866

[38] Winder T, Giamas G, Wilson PM, Zhang W, Yang D, et al.. (2013) Insulin-like growth factor receptor polymorphism defines clinical outcome in estrogen receptor-positive breast cancer patients treated with tamoxifen. Pharmacogenomics J [doi: 10.1038/tpj.2013.8]. 10.1038/tpj.2013.8 23459444

[39] Warren H, Dudbridge F, Fletcher O, Orr N, Johnson N, et al (2012) 9q31.2-rs865686 as a susceptibility locus for estrogen receptor-positive breast cancer: evidence from the Breast Cancer Association Consortium. Cancer Epidemiol Biomarkers Prev 21: 1783–91. 22859399 10.1158/1055-9965.EPI-12-0526 PMC3772723

[40] Lambrechts D, Truong T, Justenhoven C, Humphreys MK, Wang J, et al (2012) 11q13 is a susceptibility locus for hormone receptor positive breast cancer. Hum Mutat 33: 1123–32. 22461340 10.1002/humu.22089 PMC3370081

[41] Couch FJ, Gaudet MM, Antoniou AC, Ramus SJ, Kuchenbaecker KB, et al (2012) Common variants at the 19p13.1 and ZNF365 loci are associated with ER subtypes of breast cancer and ovarian cancer risk in BRCA1 and BRCA2 mutation carriers. Cancer Epidemiol Biomarkers Prev 21: 645–57. 22351618 10.1158/1055-9965.EPI-11-0888 PMC3319317

[42] Mulligan AM, Couch FJ, Barrowdale D, Domchek SM, Eccles D, et al.. (2011) Common breast cancer susceptibility alleles are associated with tumour subtypes in BRCA1 and BRCA2 mutation carriers: results from the Consortium of Investigators of Modifiers of BRCA1/2. Breast Cancer Res 13:R110 [doi: 10.1186/bcr3052]. 10.1186/bcr3052 PMC3326552 22053997

[43] Haiman CA, Chen GK, Vachon CM, Canzian F, Dunning A, et al (2011) A common variant at the TERT-CLPTM1L locus is associated with estrogen receptor-negative breast cancer. Nat Genet 43: 1210–4. 22037553 10.1038/ng.985 PMC3279120

[44] Figueroa JD, Garcia-Closas M, Humphreys M, Platte R, Hopper JL, et al (2011) Associations of common variants at 1p11.2 and 14q24.1 (RAD51L1) with breast cancer risk and heterogeneity by tumor subtype: findings from the Breast Cancer Association Consortium. Hum Mol Genet 20: 4693–706. 21852249 10.1093/hmg/ddr368 PMC3209823

[45] Stevens KN, Vachon CM, Lee AM, Slager S, Lesnick T, et al (2011) Common breast cancer susceptibility loci are associated with triple-negative breast cancer. Cancer Res 71: 6240–9. 21844186 10.1158/0008-5472.CAN-11-1266 PMC3327299

[46] Milne RL, Goode EL, García-Closas M, Couch FJ, Severi G, et al (2011) Confirmation of 5p12 as a susceptibility locus for progesterone-receptor-positive, lower grade breast cancer. Cancer Epidemiol Biomarkers Prev 20: 2222–31. 21795498 10.1158/1055-9965.EPI-11-0569 PMC4164116

[47] Fang M, Toher J, Morgan M, Davison J, Tannenbaum S, Claffey K (2011) Genomic differences between estrogen receptor (ER)-positive and ER-negative human breast carcinoma identified by single nucleotide polymorphism array comparative genome hybridization analysis. Cancer 117: 2024–34. 21523713 10.1002/cncr.25770 PMC4521590

[48] Antoniou AC, Wang X, Fredericksen ZS, McGuffog L, Tarrell R, et al (2010) A locus on 19p13 modifies risk of breast cancer in BRCA1 mutation carriers and is associated with hormone receptor-negative breast cancer in the general population. Nat Genet 42: 885–92. 20852631 10.1038/ng.669 PMC3130795

[49] Enz R (2007) The trick of the tail: protein-protein interactions of metabotropic glutamate receptors. Bioessays 29: 60–73. 17187376 10.1002/bies.20518

[50] Amanchy R, Kandasamy K, Mathivanan S, Periaswamy B, Reddy R, et al (2011) Identification of novel phosphorylation motifs through an integrative computational and experimental analysis of the human phosphoproteome. J Proteomics Bioinform 4: 22–35. 21720494 10.4172/jpb.1000163 PMC3124146

[51] Emery AC, Pshenichkin S, Takoudjou GR, Grajkowska E, Wolfe BB, et al (2010) The protective signaling of metabotropic glutamate receptor 1 is mediated by sustained, beta-arrestin-1-dependent ERK phosphorylation. J Biol. Chem 285: 26041–8. 20566651 10.1074/jbc.M110.139899 PMC2924003

[52] Chergui K, Svenningsson P, Greengard P (2005) Physiological role for casein kinase 1 in glutamatergic synaptic transmission. J Neurosci 25: 6601–9. 16014721 10.1523/JNEUROSCI.1082-05.2005 PMC6725422

[53] Sinnberg T, Menzel M, Kaesler S, Biedermann T, Sauer B, et al (2010) Suppression of casein kinase 1 alpha in melanoma cells induces a switch in beta-catenin signaling to promote metastasis. Cancer Res 70: 6999–7009. 20699366 10.1158/0008-5472.CAN-10-0645

[54] Fuja TJ, Lin F, Osann KE, Bryant PJ (2004) Somatic Mutations and Altered Expression of the Candidate Tumor Suppressors Csnk1 Epsilon, Dlg1, and Edd/Hhyd in Mammary Ductal Carcinoma. Cancer Res 64: 942–51. 14871824 10.1158/0008-5472.can-03-2100

[55] Gill S, Sargent D (2006) End points for adjuvant therapy trials: Has the time come to accept disease-free survival as a surrogate end point for overall survival? The Oncologist 11: 624–629. 16794241 10.1634/theoncologist.11-6-624

[56] Al Saleh S, Sharaf LH, Luqmani YA (2011) Signaling pathways involved in endocrine resistance in breast cancer and associations with epithelial to mesenchymal transition. Int J Oncol 38: 1197–217. 21318221 10.3892/ijo.2011.942

[57] Bostner J, Karlsson E, Pandiyan MJ, Westman H, Skoog L, et al (2013) Activation of Akt, mTOR, and the estrogen receptor as a signature to predict tamoxifen treatment benefit. Breast Cancer Res Treat 137: 397–406. 23242584 10.1007/s10549-012-2376-y PMC3539073

[58] Kirkegaard T, Witton CJ, McGlynn LM, Tovey SM, Dunne B, et al (2005) AKT activation predicts outcome in breast cancer patients treated with tamoxifen. J Pathol 207: 139–46. 16088978 10.1002/path.1829

[59] Haagenson KK, Wu GS (2010) The role of MAP kinases and MAP kinase phosphatase-1 in resistance to breast cancer treatment. Cancer Metastasis Rev 29: 143–9. 20111893 10.1007/s10555-010-9208-5 PMC4063276

